# Human epidermal growth factor receptor bispecific ligand trap RB200: abrogation of collagen-induced arthritis in combination with tumour necrosis factor blockade

**DOI:** 10.1186/ar3480

**Published:** 2011-10-07

**Authors:** Luke L Gompels, Nasser M Malik, Leigh Madden, Pei Jin, Marc Feldmann, H Michael Shepard, Ewa M Paleolog

**Affiliations:** 1 Faculty of Medicine, Kennedy Institute of Rheumatology, 65 Aspenlea Road, London, W6 8LH, Imperial College London, London, UK; 2 William Harvey Research Institute, Barts and The London School of Medicine and Dentistry, Charterhouse Square, London, EC1M 6BQ, UK; 3 Kennedy Institute of Rheumatology, University of Oxford, 65 Aspenlea Road, London, W6 8LH, UK; 4 Receptor BioLogix Inc, 3350 West Bayshore Road, Suite 150, Palo Alto, CA 94303, USA; 5 Novartis Institutes for Biomedical Research, 350 Massachusetts Avenue #1, Cambridge, MA 02139, USA; 6 Halozyme Therapeutics Inc, 11588 Sorrento Valley Road, Suite 17, San Diego, CA 92121, USA

## Abstract

**Introduction:**

Rheumatoid arthritis (RA) is a chronic disease associated with inflammation and destruction of bone and cartilage. Although inhibition of TNFα is widely used to treat RA, a significant number of patients do not respond to TNFα blockade, and therefore there is a compelling need to continue to identify alternative therapeutic strategies for treating chronic inflammatory diseases such as RA. The anti-epidermal growth factor (anti-EGF) receptor antibody trastuzumab has revolutionised the treatment of patients with EGF receptor-positive breast cancer. Expression of EGF ligands and receptors (known as HER) has also been documented in RA. The highly unique compound RB200 is a bispecific ligand trap that is composed of full-length extracellular domains of HER1 and HER3 EGF receptors. Because of its pan-HER specificity, RB200 inhibits responses mediated by HER1, HER2 and HER3 *in vitro *and *in vivo*. The objective of this study was to assess the effect of RB200 combined with TNF blockade in a murine collagen-induced arthritis (CIA) model of RA.

**Methods:**

Arthritic mice were treated with RB200 alone or in combination with the TNF receptor fusion protein etanercept. We performed immunohistochemistry to assess CD31 and *in vivo *fluorescent imaging using anti-E-selectin antibody labelled with fluorescent dye to elucidate the effect of RB200 on the vasculature in CIA.

**Results:**

RB200 significantly abrogated CIA by reducing paw swelling and clinical scores. Importantly, low-dose RB200 combined with a suboptimal dose of etanercept led to complete abrogation of arthritis. Moreover, the combination of RB200 with etanercept abrogated the intensity of the E-selectin-targeted signal to the level seen in control animals not immunised to CIA.

**Conclusions:**

The human pan-EGF receptor bispecific ligand trap RB200, when combined with low-dose etanercept, abrogates CIA, suggesting that inhibition of events downstream of EGF receptor activation, in combination with TNFα inhibitors, may hold promise as a future therapy for patients with RA.

## Introduction

Rheumatoid arthritis (RA) is a chronic autoimmune disorder characterised by severe synovial inflammation that leads to the progressive destruction of bone and cartilage. It is a severe disabling disease that affects approximately 1% of the population worldwide [1]. Despite the introduction of biological therapies such as those that target TNFα, a significant proportion of RA patients do not demonstrate a positive response to treatment. Furthermore, biologicals such as TNFα are associated with increased risk of serious infections, including tuberculosis [2-5]. In addition, the pattern of disease in patients may change over time and alternative or additional therapy may be required.

The epidermal growth factor (EGF) ligand/receptor family has been postulated to play a role in RA pathogenesis [6]. The EGF family (ErbB and the human epidermal growth factor receptor (HER)) of cell-surface receptors belong to the receptor tyrosine kinase (RTK) superfamily and consist of extracellular domains (ECDs) and an intracellular tyrosine kinase signalling domain [7,8]. The EGF family has four members, namely, EGF receptor (EGFR)/HER1/ErbB1, HER2/ErbB2, HER3/ErbB3 and HER4/ErbB4, which are activated by a large family of ligands, including EGF, as well as by transforming growth factor α (TGF-α), heparin-binding EGF-like growth factor (HB-EGF), amphiregulin (AR), β-cellulin (BTC), epiregulin (EPR), epigen (EPG) and neuregulin (NRG) [7,9,10]. Within the EGFRs there are four ECDs, with domains I and III being ligand-binding domains and domains II and IV mediating binding to each other and to other members of this receptor family. Ligand binding induces the formation of homo- or heterodimers between the receptors. For example, TGF-α and EGF bind to EGFR/HER1/ErbB1, whereas NRG4 binds to HER4/ErbB4. Depending on the dimer formed, transphosphorylation of intracellular regions occurs, leading to the activation of numerous downstream signalling pathways, which results in cell proliferation, survival and differentiation [7,9,10]. Researchers in a number of studies have suggested that the EGF ligand/receptor family has a role in the development of inflammatory arthritis [11-14]. In addition to the presence of EGF in RA synovium [6], expression of HER2/ErbB2 has been reported [12]. Other EGFR ligands in addition to EGF have been detected, namely, TGF-α and AR [11,15].

A number of agents targeting EGFRs have been successfully developed for the treatment of cancer. The first approved HER therapeutic drug, trastuzumab, is a monoclonal antibody that targets HER2 and has revolutionised the treatment of HER2-overexpressing, node-positive or node-negative breast cancer [16]. Cetuximab is a monoclonal antibody that targets HER1 and is prescribed for patients with metastatic colorectal cancer as well as for those with head and neck cancer. Similarly, panitumumab (mAb) is a fully human anti-HER1 antibody used for the treatment of metastatic colorectal cancer. In contrast, lapatinib is a RTK inhibitor which interrupts EGFR/HER1 and HER2/ErbB2 signalling and has been approved as a frontline therapy for triple-positive breast cancer and as an adjuvant therapy for patients who have progressed on trastuzumab. Erlotinib is used to treat non-small cell lung cancer and pancreatic cancer and is a RTK inhibitor targeting EGFR/HER1. Several other drugs targeting HER1, HER2 and HER3 are currently in development [17]. However, resistance to agents that target single HER family members can occur as a result of compensation by or upregulation of other HER family members [10]. Furthermore, the homodimerisation and heterodimerisation among members of the HER/ErbB family of receptors has implications for therapies directed against a single HER receptor [7].

The antagonist RB200 has been developed to target all four members of the EGFR family. RB200 is composed of the ECD of HER1/ErbB1 (amino acids 1 to 621) and HER3/ErbB3 (amino acids 1 to 621), fused with the Fc domain of human immunoglobulin G1 (IgG1) (HER1-HER3/Fc), and acts as a chimeric bispecific ligand trap. The HER3/Fc component of RB200 contains a 6×Histidine tag on the COOH terminal [18,19]. RB200 has been shown to bind both HER1/ErbB1 ligands (EGF, TGF-α, HB-EGF, AR, BTC, EPR and EPG) and HER3/ErbB3 ligands (NRG1-α and NRG1-β3) with high affinity. Moreover, RB200 inhibited EGF-stimulated and NRG1-β1-stimulated tyrosine phosphorylation of HER family proteins (HER1, HER2 and HER3) *in vitro *and has shown potency in a variety of cell proliferation assays [18]. In addition, RB200 has been shown to inhibit tumour growth *in vivo *in two human xenograft (epidermoid carcinoma and non-small cell lung cancer) nude mouse models [18]. In parallel with these findings, we have shown that adenoviral delivery of a human EGFR family inhibitor, herstatin, significantly abrogated all clinical signs of collagen-induced arthritis (CIA) in mice [20]. Herstatin is an alternative splice variant of HER2/ErbB2 that retains intron 8, which results in the formation of an approximately 68 kDa protein. This protein disrupts dimerisation and serves as a natural inhibitor of native HER2/ErbB2 as well as HER1/ErbB1 and HER3/ErbB3 [21]. Thus, in addition to being well-validated targets for cancer therapy, HER-targeted treatments may have therapeutic potential across a range of autoimmune and inflammatory conditions, including RA.

In the present study, we explored the therapeutic effects of RB200 in combination with the TNFα inhibitor etanercept in murine CIA as a model of human RA. Our data demonstrate that RB200 potently reduced all measures of arthritis in CIA. Furthermore, when low-dose RB200 and etanercept were combined, there was complete abrogation of CIA. This was confirmed on the basis of *in vivo *E-selectin-targeted fluorescence imaging, which is a novel technique that we have shown can detect endothelial activation in CIA and delineate subclinical arthritis [22]. These data suggest that targeting EGFR-mediated responses in combination with TNFα may provide additional clinical benefit in patients with RA.

## Materials and methods

### Induction and assessment of arthritis

Type II collagen was extracted from bovine articular cartilage as previously described [19,20,23]. For induction of CIA, 12-week-old male DBA/1 (H-2^q^) mice (Harlan UK Ltd, Oxon, UK) received a single intradermal injection at the base of the tail of 100 μg of bovine type II collagen emulsified in complete Freund's adjuvant and *Mycobacterium tuberculosis *H37 Ra (Difco Becton Dickinson, Cowley, Oxford, UK). The animal work was conducted under Home Office Project Licence PPL 70/5446, 'Pathogenesis and therapy for RA', under the operatives of the Animals (Scientific Procedures) Act 1986.

The incidence of arthritis in each group of animals reached at least 80%. The earliest signs of disease were observed on day 14 after immunisation, with day 27 postimmunisation being the median day of arthritis onset. The first clinical signs of arthritis were considered to be present when oedema and/or erythema involving any one of the four paws was observed. Human TNF type II receptor fusion protein (etanercept) was obtained from Wyeth Pharmaceuticals, Taplow, UK, and RB200 was a gift from Dr Michael Shepard (Halozyme Therapeutics, San Diego, CA, USA). Control animals received equivalent volumes of PBS. The mice were treated intraperitoneally on the day of disease onset (day 1) and then on days 4 and 7 of disease with RB200 at the indicated doses. Control mice received intraperitoneal injections of either etanercept or equivalent volumes of PBS. The study involved four comparable experiments to test the efficacy of different doses of RB200. All treatments were administered in a blinded fashion.

Paw swelling was measured with 0- to 10-mm callipers (Kroeplin, Schluchern, Germany). Each limb was further assigned a clinical score as follows: 0 = normal paws and no clinical features of inflammation, 1 = slight oedema or erythema, 2 = pervading oedema or erythema involving the entire paw and 3 = pronounced oedema and erythema leading to incapacitated limb mobility.

### Histological analysis

Hind paws were removed postmortem and sliced along the sagittal plane prior to fixation with 1% paraformaldehyde. Paws were decalcified for 14 days in 0.3 M ethylenediaminetetraacetic acid, and CD31 expression was examined by immunohistochemistry using anti-mouse CD31 antibody (MEC13.3 rat anti-mouse mAb; Pharmingen, San Diego, CA, USA). The sections were then incubated with biotinylated rabbit anti-rat antibody (1:400 dilution; Dako, Ely, UK), followed by avidin-biotin-peroxidase complex and diaminobenzidine-H_2_O_2_. Immunostained sections were counterstained with Harris's haematoxylin. As a negative control, rat IgG2a antibody was used (R35-95; Pharmingen). Scores were graded in a blinded fashion, and the criteria assessed were (1) proximity of CD31 staining to the joint, (2) strength of CD31 staining, (3) degree of infiltration of synovial cells into the joint and (4) overall status of joint architecture. Sections were further stained with haematoxylin and eosin as well as with toluidine blue. Each stained section was scored by a blinded observer for changes in the joint architecture using our previously described grading system [20,24] as follows: 0 = normal, 1 = minimal synovitis and bone erosion limited to discrete foci, 2 = extensive synovial hyperplasia and some destruction of segments cartilage and bone and 3 = complete destruction of the joint architecture.

### Fluorescence optical imaging *in vivo*

Anti-mouse E-selectin mAb (MES-1) was purified from a hybridoma cell line (a kind gift from Dr Derek Brown, UCB Celltech, Slough, UK) and conjugated to DyLight 750 near-infrared (NIR) fluorophore according to the manufacturer's instructions (Thermo Scientific, Loughborough, UK). For imaging studies, mice were anaesthetised with a mixture of isofluorane and oxygen prior to intravenous injection of 5 μg of antibody. Optical imaging was performed using a Kodak FX Pro multispectral fluorescence reflectance imaging device (Carestream Molecular Imaging, Rochester, NY, USA). Filters for excitation and emission were set to 730 nm and 770 nm, respectively. An identical imaging protocol (exposure time 60 seconds, 4× binning, F-stop 2.51, field of view 158.5, focal plane 9.4 mm) was maintained for all images as previously described [22].

Images were analysed by a blinded observer using the Kodak Molecular Imaging version 5.0 software program (Carestream Health, Rochester, NY, USA). A region of interest (ROI) created by an automated tool was determined around the inflamed or control paw. Mean ROI signal intensities are expressed as arbitrary fluorescent signal intensity units. Fluorescence greyscale images were artificially coloured for depiction purposes according a colour scale set to the highest and lowest levels of mean fluorescence intensity (MFI) (with red and purple indicating maximum and minimum light intensity, respectively). The fluorescence images were coregistered onto X-ray images taken concurrently.

### Statistical analyses

The data were analysed using GraphPad Prism version 5.01 software (GraphPad Software, Inc, San Diego, CA, USA) using one-way analysis of variance (ANOVA) or two-way ANOVA as appropriate. Histological data were analysed by Χ^2 ^test for trends.

## Results

**Figure 1:**
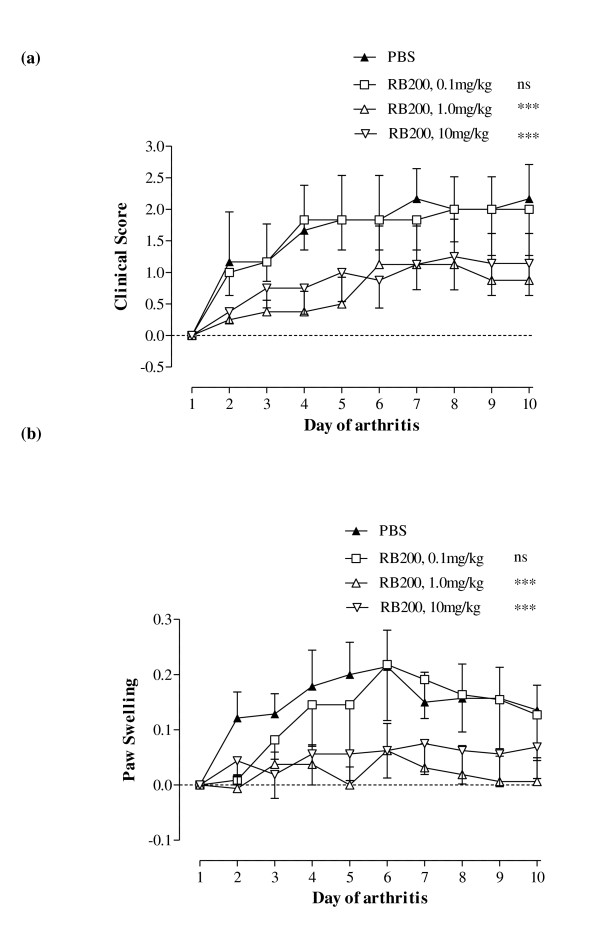
**Dose-dependent effect of RB200 in collagen-induced arthritis**. Following the onset of arthritis induced by bovine collagen, mice were treated intraperitoneally on the day of disease onset (day 1) and then on days 4 and 7 of disease with RB200 at a dose of 0.1 mg/kg (*n *= 6), 1 mg/kg (*n *= 8) or 10 mg/kg (*n *= 8). Control mice received an equivalent volume of PBS intraperitoneally (*n *= 6). **(a) **The increase in clinical score was assessed over a 10-day period, and the data are presented as means ± SEM compared to day 1 of arthritis. **(b) **Paw swelling was measured with callipers, and data are means ± SEM of both hind paws expressed expressing the difference from baseline (in millimetres). The data were derived from a representative experiment and analysed using two-way analysis of variance vs PBS-treated mice. ns = not significant; ***P < 0.001.

Treatment of CIA with RB200 dose-dependently inhibited the increase in clinical score (Figure 1a) and paw swelling (Figure 1b). The mice were treated intraperitoneally on the day of disease onset (day 1) and then on days 4 and 7 of disease. RB200 at a dose of 0.1 mg/kg was ineffective, but 1 mg/kg and 10 mg/kg provided a significant benefit (*P *< 0.001 vs PBS for both paw swelling and change in clinical score). On day 10 of established disease, the mean increase in clinical score ± SEM from day 1 of arthritis in mice treated with either PBS or RB200 at 0.1, 1 and 10 mg/kg were 2.17 ± 0.54, 2.00 ± 0.73, 0.88 ± 0.74 and 1.14 ± 0.51, respectively (Figure 1a). There was also a reduction in hind paw swelling in RB200-treated mice (Figure 1b).

**Figure 2:**
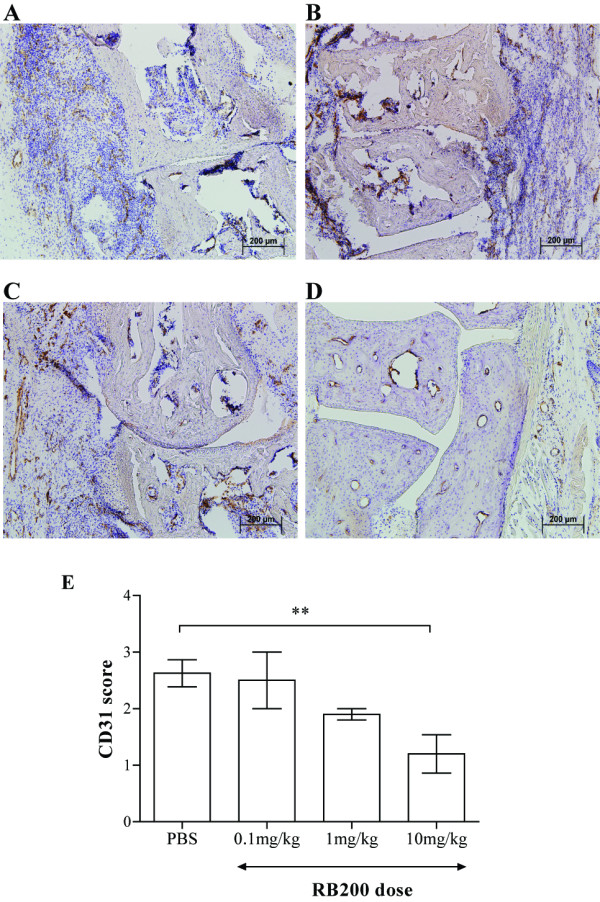
**RB200 reduces synovial vascularity in collagen-induced arthritis**. Following the onset of arthritis induced by bovine collagen, mice were treated intraperitoneally on the day of disease onset (day 1) and then on days 4 and 7 of disease with RB200 at a dose of 0.1 mg/kg (*n *= 6), 1 mg/kg (*n *= 8) or 10 mg/kg (*n *= 8). Control mice received an equivalent volume of PBS intraperitoneally (*n *= 6). Representative CD31-stained sections from the first metatarsal joints of mice are shown. **(a) **PBS treatment. **(b) **0.1 mg/kg RB200. **(c) **1 mg/kg RB200. **(d) **10 mg/kg RB200. **(e) **CD31-stained sections were scored subjectively in a blinded fashion. Scale bars are indicated. The criteria assessed included proximity of positive staining to the joint, strength of immunopositive staining and overall status of joint architecture. Statistical analyses were carried out using one-way analysis of variance vs PBS-treated mice. **P < 0.01.

Mouse paws were taken on day 10 of arthritis, and frozen sections were cut and stained with anti-CD31 antibody. For each mouse, the metatarsal-tarsus joints of both paws were examined, and representative images from RB200- and PBS-treated mice are shown in Figures 2a through 2d. Joint sections of PBS-treated mice showed high numbers of infiltrating cells in the inflamed synovium, together with invasion and erosion of bone by the synovium, associated with marked CD31 expression. In contrast, joints from mice treated with 1 or 10 mg/kg RB200 were relatively protected, with generally normal appearance, well-preserved joint architecture and relatively few CD31-positive blood vessels. In addition, an overall subjective score between 0 and 3 was used to grade CD31-immunopositive staining. Analysis of mean scores for each treatment group showed there was a significant reduction in CD31 immunopositivity in mice treated with 10 mg/kg RB200 (*P *< 0.01) (Figure 2e).

**Figure 3:**
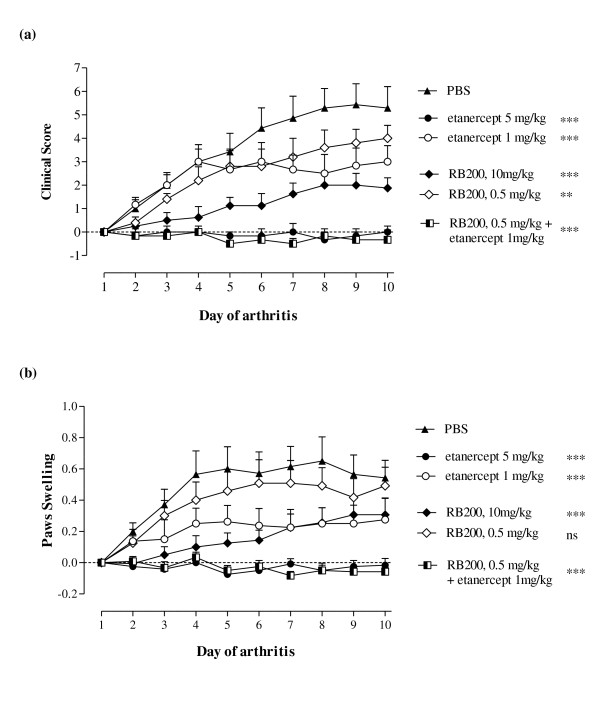
**Additive effect of low-dose etanercept and RB200 in collagen-induced arthritis**. Following the onset of arthritis induced by bovine collagen, mice were treated intraperitoneally on the day of disease onset (day 1) and then on days 4 and 7 of disease with RB200 at a dose of 0.5 mg/kg (*n *= 6) or 10 mg/kg (*n *= 8) or with a combination of RB200 0.5 mg/kg and etanercept 1 mg/kg (*n *= 6). Control mice received 5 mg/kg etanercept (*n *= 6) or 1 mg/kg etanercept (*n *= 7) or equivalent volumes of PBS (*n *= 7) intraperitoneally. **(a) **The increase in clinical score was assessed over a 10-day period, and data are presented as means ± SEM compared to day 1 of arthritis. **(b) **Paw swelling was measured with callipers, and data are presented as means ± SEM of both hind paws expressed as the difference from baseline (in millimetres). The data were derived from a representative experiment and analysed using two-way analysis of variance vs PBS-treated mice. ns = not significant; **P < 0.01; ***P < 0.001.

Importantly, when a low dose of RB200 was combined with a suboptimal dose of the TNFα inhibitor etanercept (also given on days 1, 4 and 7 of arthritis), a marked beneficial response was observed, with the combination of 0.5 mg/kg RB200 and 1 mg/kg etanercept having a marked effect on both measures of disease activity in CIA (Figure 3). In this experiment, 0.5 mg/kg RB200 alone had no effect on paw swelling (*P *> 0.05 vs PBS-treated mice) and only a modest effect on clinical score (*P *< 0.01), whereas 10 mg/kg RB200 significantly, albeit not completely, reduced both parameters, in agreement with the experiment illustrated in Figure 1. Similarly, 1 mg/kg etanercept alone partially reduced clinical score and paw swelling (*P *< 0.001) (Figure 3). In contrast, the combination of low doses of RB200 and etanercept was significantly different from either low-dose etanercept alone (*P *< 0.001 for both clinical score and paw swelling) or low-dose RB200 alone (*P *< 0.001 for both clinical score and paw swelling). The effectiveness of the combination of 0.5 mg/kg RB200 and 1 mg/kg etanercept was comparable to that seen with the optimal dose of etanercept (5 mg/kg).

**Figure 4:**
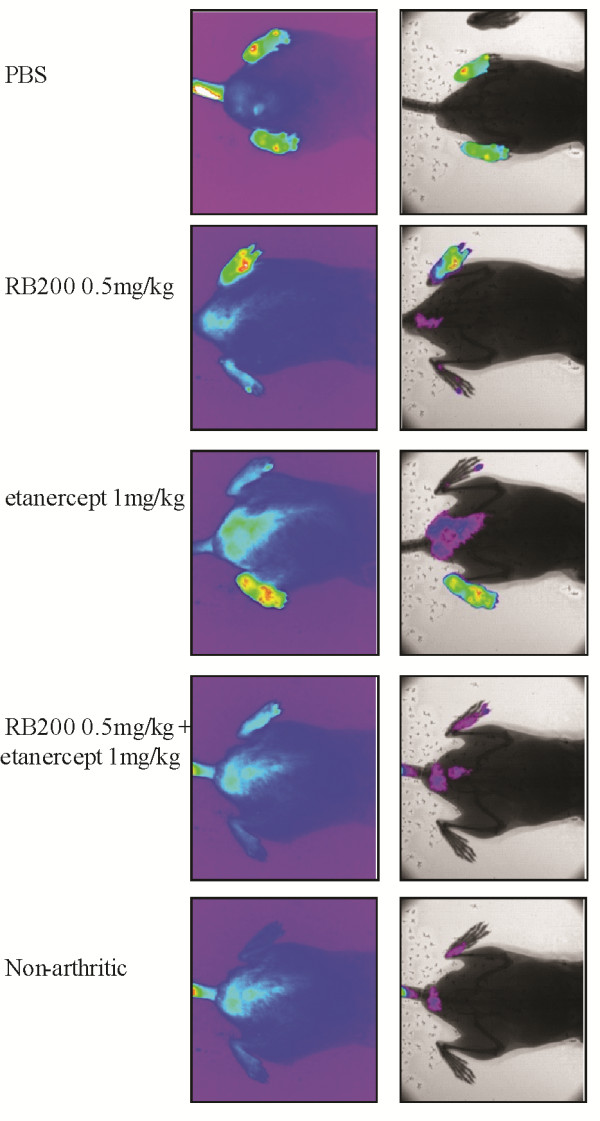
***In vivo *E-selectin-targeted fluorescence imaging in collagen-induced arthritis following treatment with low dose etanercept and/or RB200**. Following the onset of arthritis induced by bovine collagen, mice were treated intraperitoneally on the day of disease onset (day 1) and then on days 4 and 7 of disease with RB200 at a dose of 0.5 mg/kg or 1 mg/kg etanercept or with a combination of 0.5 mg/kg RB200 and 1 mg/kg etanercept. Control mice received equivalent volumes of PBS intraperitoneally. For imaging, mice were injected intravenously with near-infrared dye-labelled anti-E-selectin antibody (5 μg in 200 μl). Images were obtained eight hours after injection of the dye-antibody conjugate. Left panels show representative fluorescent images obtained after application of a colour wheel to depict signal intensity. Right panels show corresponding images coregistered with X-rays after subtraction of background fluorescence.

We further applied *in vivo *optical imaging to investigate the effect of RB200. Figure 4 demonstrates representative examples of fluorescence imaging with NIR dye-labelled anti-E-selectin antibody for representative mice. The images were coregistered with plain digital X-rays of arthritic paws. E-selectin-specific fluorescent signals were detected in both hind paws of animals with CIA (PBS-treated), but not in healthy (nonarthritic) mice. Fluorescent signals were also visible in the arthritic paws of mice that received low-dose etanercept alone or low-dose RB200 alone. These data further demonstrate that the combination of low-dose etanercept and RB200 has a marked effect in decreasing the E-selectin-targeted signal in animals with arthritis compared with either treatment on its own. Although both low-dose etanercept (1 mg/kg) and RB200 (0.5 mg/kg) had some overall therapeutic efficacy, it was only with combination treatment that there was complete abrogation of fluorescent signal.

**Figure 5:**
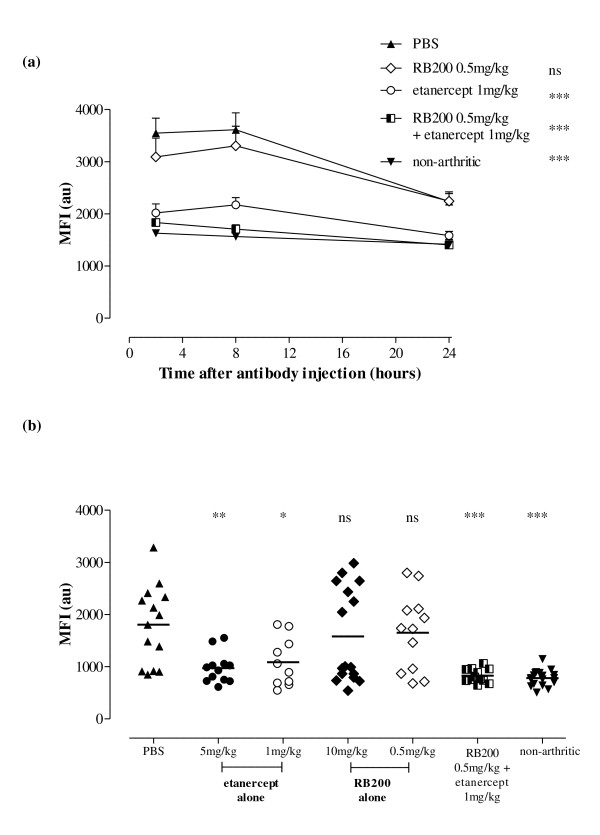
**Additive effects of low-dose etanercept and RB200 on E-selectin-targeted *in vivo *fluorescent imaging in collagen-induced arthritis**. Following the onset of arthritis induced by bovine collagen, mice were treated intraperitoneally on the day of disease onset (day 1) and then on days 4 and 7 of disease with RB200 at a dose of 0.5 mg/kg (*n *= 6) or 10 mg/kg (*n *= 8) or with a combination of RB200 0.5 mg/kg and etanercept 1 mg/kg (*n *= 6). Control mice received 5 mg/kg etanercept (*n *= 6), 1 mg/kg etanercept (*n *= 7) or equivalent volumes of PBS (*n *= 7) intraperitoneally. For imaging, mice were injected intravenously with near-infrared dye-labelled anti-E-selectin antibody (5 μg in 200 μl). Images were obtained 2, 8 and 24 hours after injection of the dye-antibody conjugate. **(a) **The results are expressed in arbitrary units (au) of mean fluorescence intensity (MFI) ± SEM for both paws and were analysed using two-way analysis of variance (ANOVA) vs PBS-treated mice. ns = not significant; ***P < 0.001. For clarity, data derived from animals treated with 10 mg/kg RB200 or 5 mg/kg etanercept were omitted. **(b) **Data from a representative experiment are presented and expressed in arbitrary units (au) of mean fluorescence intensity (MFI) ± SEM for individual paws eight hours after injection of the dye-antibody conjugate. They were analysed using one-way ANOVA vs PBS-treated mice. ns = not significant; *P < 0.05; **P < 0.01; ***P < 0.001.

Quantification of MFI over 24 hours in the different treatment groups is shown in Figure 5. For the purposes of clarity, the MFI signal returned from animals treated with either high-dose etanercept or RB200 are not included in Figure 5a but are included in Figure 5b. These data show that combination treatment returned the anti-E-selectin MFI to the levels seen in healthy animals that were not immunised against CIA (no significant difference by two-way ANOVA) (Figure 5a). Indeed the effect of combination treatment was significantly different from that seen with either low-dose etanercept alone (*P *< 0.01 by two-way ANOVA) or low-dose RB200 alone (*P *< 0.001 by two-way ANOVA). In a prior study we have demonstrated that E-selectin-targeted fluorescence imaging is able to detect subclinical levels of fluorescence in animals with a clinical score of 0 [22]. Here combination therapy involving low-dose RB200 plus low-dose etanercept abrogated fluorescent signal in all imaged paws to levels seen in healthy controls, whereas following treatment with either high-dose RB200 or etanercept, increased signal levels were evident in some of the paws (Figure 5b).

**Figure 6:**
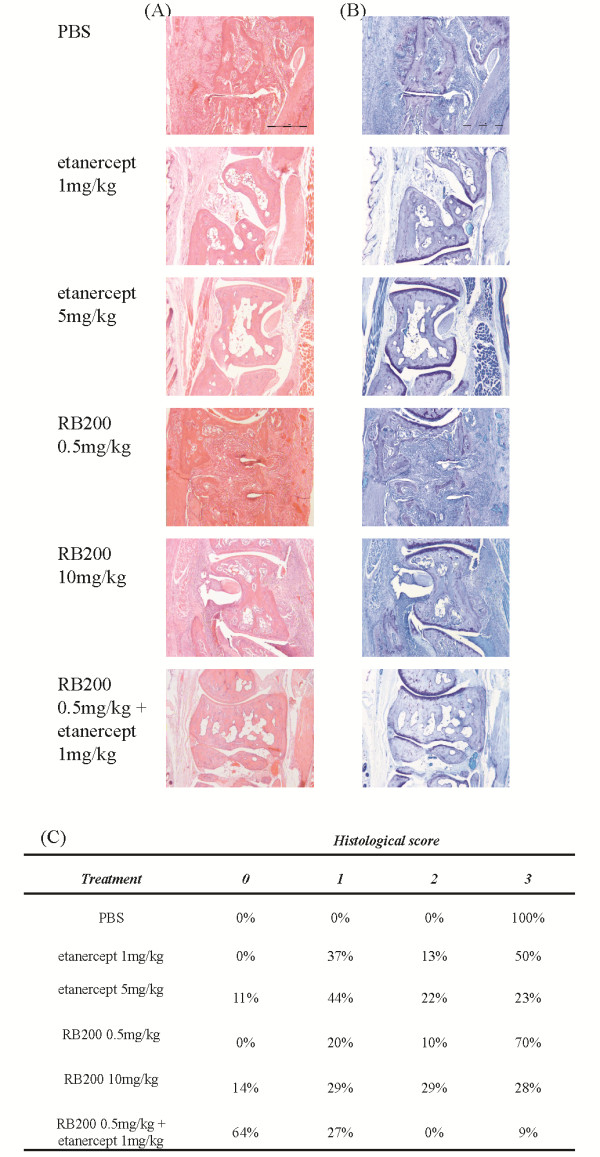
**A combination of low-dose etanercept and RB200 results in normalisation of joint architecture in collagen-induced arthritis**. To assess damage to cartilage and joints, we stained serial sections of hind feet (4 μm) with **(a) **haematoxylin and eosin or **(b) **toluidine blue. Tibiotarsal joint sections from animals representing the median score for each group are shown. Bars = 100 μm. **(c) **Percentage of sections exhibiting normal appearance (score 0), minimal synovitis and bone erosion (score 1), extensive synovial hyperplasia and some destruction of cartilage and bone (score 2), and complete destruction of the joint architecture (score 3). Data are derived from 9 to 14 sections per group.

These data are supported by histological findings. Using serial sections stained with either haematoxylin and eosin or toluidine blue, we evaluated tibiotarsal joints for signs of synovitis and erosive changes to bone and cartilage. There were was a dose-dependent effect of both RB200 alone and etanercept alone on joint architecture, with progressively fewer severely destroyed joints and more joints with mild or moderate destruction (Figure 6). However, the most pronounced effect was observed when the combination treatment was used, with 64% joints appearing normal (score 0; *P *< 0.001 vs untreated by Chi squared test for trend) compared to 0% in mice that received low-dose RB200 alone (*P *> 0.05 vs untreated and *P *< 0.001 vs combination treatment) and 0% in mice treated with low-dose etanercept alone (*P *< 0.05 vs untreated and *P *< 0.01 vs combination treatment). Indeed the combination treatment was even more effective than high-dose etanercept alone (*P *< 0.05 vs combination treatment), highlighting the effectiveness of combined pan-EGFR and TNFα-targeted treatment in promoting joint protection.

## Discussion

Anti-HER2 antibody trastuzumab has revolutionised the treatment of patients with EGFR-positive breast cancer, and agents targeting EGFR/HER1 are in use for colorectal cancer therapy as well as for head and neck cancer. Interestingly, researchers in a number of studies have suggested that the family of EGF ligands and receptors may have a role in the development of RA. For example, expression of one of the primary ligands for EGFR/HER1, TGF-α, is increased at both the mRNA and protein levels in RA synovium [11]. High levels of another EGFR ligand, EGF, in RA synovial fluids have also been demonstrated. However, researchers in another study reported increased expression of AR, but not EGF, ERG, EPG, BTC, NRG1 or HB-EGF, at the mRNA level in RA compared to OA tissue. However, the reason for the discrepancy between this report and other studies is unclear [15]. Expression of HER2/ErbB2 in the synovium of RA patients has been described further [11,12]. A treatment modality targeting EGFRs might therefore be an attractive option in immunotherapy for RA.

RB200 is a heterodimer of the HER1/ErbB1 and HER3/ErbB3 ECD [18,25]. This prototypical bispecific ligand trap binds HER1/ErbB1 and HER3/ErbB3 ligands, inhibits proliferation of a broad spectrum of cultured cancer cells and suppresses growth of tumour xenografts in mouse models [18]. The efficacy of RB200 has been investigated previously in other *in vivo *models. RB200 inhibited EGF- and NRG1-β1-stimulated tyrosine phosphorylation of HER1, HER2 and HER3 and also showed potency in cell proliferation assays [18]. In addition, RB200 has been shown to inhibit tumour growth *in vivo *in two human tumour xenograft (epidermoid carcinoma and non-small cell lung cancer) nude mouse models [18]. Thus, in contrast to antibodies such as trastuzumab, RB200 has the potential to inhibit signalling by multiple EGFR ligands. In this study, we examined the effect of RB200 on established CIA, which has been widely used for *in vivo *testing of candidate therapeutics for RA, such as TNFα inhibitors [26,27] and inhibitors of angiogenesis [23,28,29]. Moreover, we have previously reported that adenoviral delivery of the human EGFR family inhibitor herstatin significantly abrogated murine CIA [20], demonstrating that by blocking signalling downstream of HER2/ErbB2, HER3/ErbB3 was effective in CIA. In the current study, administration of RB200 produced a significant dose-dependent reduction in disease severity in CIA. Interestingly, RB200 showed effective therapeutic activity at doses as low as 1 mg/kg when administered three times in the 10-day period of arthritis. Indeed 1 mg/kg was not significantly different from 10 mg/kg (by two-way ANOVA), and higher doses of RB200 up to 15 mg/kg were no more effective (not shown). EGF has been reported to promote angiogenesis [30,31], and there was also a significant reduction of CD31-immunopositive staining in RB200-treated mice, reflecting a reduction in synovial vessels. In comparison to controls, there was also normalisation of joint architecture in arthritic mice treated with RB200. These data suggest that inhibition of EGFR-mediated responses is of potential use for the treatment of RA.

The efficacy of combination treatment with low-dose etanercept and RB200 is of potential interest. A significant proportion of patients with RA do not respond to TNFα blockade, and therefore there is a compelling need to continue identification of alternative therapeutic strategies for chronic inflammatory diseases such as RA. Nonetheless, biological therapies, particularly those targeting TNFα, are an undoubted success in RA, and hence an approach combining TNFα inhibition with, for example, an inhibitor of angiogenesis or with an inhibitor of EGFR-induced events might be useful. Moreover, targeting such separate mechanistic pathways could be beneficial, unlike the combination of targeting IL-1 and TNFα, which has been shown to be proinfective [25]. In the present report, we demonstrate that low doses of the TNFα inhibitor etanercept plus RB200, though only modestly effective alone, completely abolished CIA to levels comparable to those observed following treatment with an optimal dose of etanercept.

We have also previously described the use of fluorescently labelled mAb against E-selectin to localise endothelial activation in inflamed tissues *in vivo *and have found this approach to be a sensitive, specific and quantifiable molecular imaging technique in CIA [22]. The combination of low doses of etanercept and RB200 resulted in complete abrogation of E-selectin-targeted fluorescent signal to levels seen in healthy controls and was more effective than 5 mg/kg etanercept. In fact, mice treated with 5 mg/kg etanercept displayed greater variability of MFI values, implying that disease activity may not have been completely abolished. We have previously shown a degree of fluorescent signal in mouse paws that had been given a clinical score of 0, suggesting that fluorescence imaging may be more sensitive than conventional scoring [22]. Imaging of mice treated with 5 mg/kg etanercept for 2 to 24 hours did in fact reveal that the MFI readings were significantly different (*P *< 0.01) from those in nonarthritic mice (not shown), whereas MFI readings for mice treated with etanercept plus RB200 were not significantly different from nonarthritic mice but lower than those treated with either low-dose etanercept alone (*P *< 0.01 by two-way ANOVA) or low-dose RB200 alone (*P *< 0.001 by two-way ANOVA). The *in vivo *fluorescence imaging data are supported by observations of normalised joint architecture in animals treated with etanercept plus RB200.

Our findings substantiate the hypothesis that combination treatment with low doses of etanercept and RB200 has a significant beneficial effect in CIA, although there are a number of shortcomings of this study, the most notable being the absence of mechanism of action studies to elucidate the mode of action of RB200 in CIA. In terms of a potential *in vivo *mechanism of action of RB200, angiogenesis is a key feature of RA [32], and indeed EGF has been reported to promote angiogenesis [30,31,33], in part through production of proangiogenic factors such as VEGF, IL-8 and basic fibroblast growth factor [13,30,34,35]. Direct angiogenic effects have also been demonstrated in human microvascular endothelium in response to EGF or TGF-α [36-38]. Other EGFR ligands, such as NRG1-β3, have been reported to promote angiogenesis [39], and targeting EGFRs, alone or in combination with VEGF inhibition, has been shown to reduce angiogenesis in *in vivo *models [38,40,41]. Activation of EGFR signalling may also contribute to synovial hyperplasia in CIA, which is also a major pathological feature of RA that precedes clinical presentation and prevails throughout the disease [31]. This is suggested by a report that trastuzumab, which binds the HER2/ErbB2 receptor, inhibits RA synovial cell growth [12]. RB200 has the potential to inhibit responses to multiple EGFR ligands in CIA. Although inhibition of synovial angiogenesis or proliferation was not directly addressed in the present study, which is clearly a limitation in terms of understanding a possible *in vivo *mode of action, studies assessing whether synovial cell responses might be affected by RB200 are in progress. However, inhibition of synovial angiogenesis by RB200 could be implicated by the reduction in CD31-positive blood vessels, which we have demonstrated in CIA. Furthermore, EGF, when combined with proinflammatory mediators such as TNFα and IL-1, has been shown to increase expression by fibroblasts of matrix metalloproteinases (MMPs) such as MMP-1, MMP-3 and MMP-13 [42,43] and to upregulate fibroblast proliferation [44]. Moreover, these responses were abrogated by a small-molecule inhibitor of HER2/ErbB2. It would be useful to examine whether RB200 modulates RA fibroblast responses *in vitro*, in particular by assessing MMP expression, as this would provide invaluable insight into how RB200 might affect CIA.

It is also unclear which of the many EGFR ligands RB200 inhibits in CIA. In addition to the presence of EGF in RA synovium [6], expression of TGF-α and AR has been described in RA [11,15], and RB200 has been reported to bind to all of these ligands [18]. Interestingly, the pan-EGFR activity of RRB200 suggests that this molecule could offer additional benefit over approved treatments such as trastuzumab (a mAb that targets HER2) [45], cetuximab and panitumumab (anti-EGFR/HER1 antibodies), as well as inhibitors such erlotinib, lapatinib and gefitinib [46-48]. Resistance to these agents can occur as a result of coexpression and ligand activation of other HER family members [49-51]. Furthermore, homodimerisation and heterodimerisation among members of the ErbB receptors is known to occur [10], hence therapies directed against a single HER receptor are likely to be less effective [7], suggesting that agents such as RB200, which target multiple receptors, are desirable. Indeed the pleiotropic effects mediated by ligation of the EGFR family (for example, angiogenesis, inflammation and synovial invasion) and the expression of multiple EGFR ligands in RA suggest that pan-EGFR blockade is likely to have a broad beneficial effect in arthritis.

## Conclusions

The studies described in this report demonstrate that the potent human EGFR HER1/HER3 ECD heterodimer RB200 has a significant abrogating effect in a mouse model of RA, namely, CIA. A recent case report described a patient with long-standing RA who had previously been treated with rituximab and adalimumab. This patient experienced a significant reduction in joint pain during treatment with anti-EGFR/HER1 antibody cetuximab for head and neck cancer [52]. Together with our data in murine CIA, these findings highlight the usefulness of targeting the EGFR family in RA.

However, cellular heterogeneity, redundancy of molecular pathways and effects of the microenvironment all likely contribute to the disease heterogeneity seen in CIA. This makes it unlikely that therapy directed at one abnormally activated signalling pathway, such as that downstream of EGFR activation, is likely to be sufficient [53]. Therefore, combined blockade of functionally linked and relevant multiple targets has become an attractive therapeutic strategy, particularly given the increased risk of infection associated with some classes of biologicals [54-56]. Combination of RB200 with the TNFα blocker etanercept resulted in complete abrogation of arthritis, suggesting that HER-targeted therapies developed for the treatment of cancer may also be effective in RA.

## Abbreviations

ANOVA: analysis of variance; AR: amphiregulin; BTC: β-cellulin; CIA: collagen-induced arthritis; EGF: epidermal growth factor; EPG: epigen; EPR: epiregulin; HB-EGF: heparin-binding epidermal growth factor-like growth receptor; HER: human epidermal growth factor receptor; IL: interleukin; mAb: monoclonal antibody; MAPK: mitogen-activated protein kinase; MMP: matrix metalloproteinase; NIR: near-infrared; NRG: neuregulin; PBS: phosphate-buffered saline; RA: rheumatoid arthritis; RTK: receptor tyrosine kinase; TGF-α: transforming growth factor α; TNF: tumour necrosis factor; VEGF: vascular endothelial growth factor.

## Competing interests

PJ and HMS are former employees of Receptor BioLogix Inc and declare competing financial interests. MF and EMP have acted as consultants for Receptor BioLogix Inc. The other authors declare that they have no competing interests.

## Authors' contributions

HMS designed the study. PJ assisted in the study design. LLG, LM and NMM performed all the experiments, data analysis and drafting of the manuscript. EMP and MF assisted in the study design and coordination and oversaw the data analysis and drafting of the manuscript. All authors read and approved the final manuscript for publication.
